# Study of SARS-CoV-2 Spike Protein Wild-Type and the Variants of Concern Real-Time Interactions with Monoclonal Antibodies and Convalescent Human Serum

**DOI:** 10.3390/bios13080784

**Published:** 2023-08-03

**Authors:** Ieva Plikusiene, Vincentas Maciulis, Silvija Juciute, Arunas Ramanavicius, Almira Ramanaviciene

**Affiliations:** 1 NanoTechnas—Center of Nanotechnology and Materials Science, Faculty of Chemistry and Geosciences, Vilnius University, Naugarduko str. 24, LT-03225 Vilnius, Lithuania; 2 State Research Institute Center for Physical and Technological Sciences, Sauletekio ave. 3, LT-10257 Vilnius, Lithuania

**Keywords:** SARS-CoV-2, spectroscopic ellipsometry, quartz crystal microbalance with dissipation, immunosensor, kinetics

## Abstract

The spike (S) protein and its receptor-binding domain (RBD) of the coronavirus SARS-CoV-2 have been continually evolving, yielding the majority of significant missense mutations and new variants of concern. In this study, we examined how monoclonal antibodies against RBD (mAbs-SCoV2-RBD) and polyclonal antibodies present in convalescent human serum specifically interact with the S protein of wild-type and SARS-CoV-2 variants of concern (VOCs) in real time and how this can be reflected through surface mass density. Moreover, we combined two distinct, label-free measurement techniques: one based on changes in surface electromagnetic waves after reflection from the surface, and the other on changes in acoustic waves. The results demonstrated that dry surface mass density (Γ^SE^) of mAbs-SCoV2-RBD attached to the RBD of the S protein decreases three-fold, from 148 ng/cm^2^ to 46 ng/cm^2^, due to the B.1.351 or so-called beta mutation of coronavirus and its S protein (SCoV2-β). Consequently, the obtained wet mass Γ^QCM-D^ resulted in values two times lower, from 319 ng/cm^2^ to 158 ng/cm^2^, and the hydration of mAbs-SCoV2-RBD/SCoV2-β immune complex was 70.88%. Conversely, when polyclonal antibodies present in convalescent human serum form immune complexes with the S protein of SARS-CoV-2 variants of concern, the Γ^SE^ decreased from 279 ng/cm^2^ to 249 ng/cm^2^, and Γ^QCM-D^ from 1545 ng/cm^2^ to 1366 ng/cm^2^. These results can give insights into the differences between the interaction of monoclonal and polyclonal antibodies with SARS-CoV-2 VOCs.

## 1. Introduction

During the pandemic, various ways of detecting SARS-CoV-2 and diagnosing COVID-19 infection were developed, including different tests capable of detecting viral antigens or specific antibodies [1,2]. The methods that are commonly applied to study the biomolecules’ real-time interaction, including antigen–antibody immune complex formation, are the following: microscale thermophoresis (MST), isothermal titration calorimetry (ITC), biolayer interferometry (BLI), and surface plasmon resonance (SPR) [3,4,5,6]. Non-linear optics, machine learning technologies, and theoretical modeling can be used to improve optical biosensing [7,8]. Various optical biosensors based on plasmonic effects were developed for the effective detection of viral proteins from exhaled air, nasopharyngeal swabs, and saliva [7,9,10]. Some of these methods, such as SPR and BLI, have been successfully applied for the biosensing and characterization of antibodies’ interaction with SARS-CoV-2 structural proteins, or for the assessment of the formed immune complexes’ stability and for the evaluation of antibody affinity [11,12]. However, there are some limitations worth mentioning; for instance, MST does not provide information on interaction kinetics and requires labeling; ITC uses a large volume of samples, takes time, and is not always capable of providing real-time dynamic measurements [13]. Highly sensitive, non-contact, label-free methods capable of monitoring immune complex formation on the surface in real time are in high demand for extensive examination of antigen and antibody interactions. Recently, surface-sensitive methods such as spectroscopic ellipsometry (SE) based on electromagnetic waves and quartz crystal microbalance with dissipation (QCM-D) based on acoustic waves attracted attention to studies of the formation of immune complexes in real time [2,14,15,16]. QCM was successfully applied for the label-free detection of antibodies against the phospholipase A2 receptor, and for the characterization of monoclonal antibodies against the hepatitis B virus [17,18]. Moreover, QCM-D was also applied to the time-resolved study of anti-BSA detection [19]. The SE is a non-destructive, label-free, and extremely sensitive optical method that is able to determine two ellipsometric parameters during one measurement: Ψ, which corresponds to the light wave amplitude, and Δ, which provides information on the light phase shift upon reflection from the sample. Moreover, after data analysis, SE allows the determination of the protein layers’ thicknesses and dielectric constants, which can further be used to calculate the surface mass density of the immobilized protein [20,21,22]. Therefore, the evaluation of these unique properties requires regression analysis. The other method that is based on acoustic waves and enables the study of antibody and antigen interaction in real time without labeling is QCM-D. It allows the determination of the density of surface mass attached to a quartz crystal sensor surface, as well as the viscoelastic properties of the protein layer, by measuring the frequency shifts (ΔF) and energy dissipation (ΔD) of vibrational resonance overtones [23,24]. In the SE and QCM-D methods, response signals depend on the immobilized protein mass, but SE provides additional information about the dielectric properties of the protein layer, while QCM-D provides information about the viscoelastic properties. Simultaneously combining SE and QCM-D in one real-time measurement of antigen–antibody interaction kinetics can provide information on the solvent content of thin protein layers formed on the surface [24]. The optical methods do not measure surface mass directly; they are able to measure changes in the refractive index. Moreover, the optical techniques are not able to obtain the coupled solvent between the protein molecules in the formed layers. Furthermore, optical methods cannot quantify structural rearrangements of molecular layers. As QCM-D is an acoustic waves method, it can be combined with optical techniques to obtain complementary data about the mass and structural features of the layer. The hybrid SE/QCM-D approach may disclose further new possibilities for antigen–antibody interaction and immune complex formation analysis, delivering quantitative information that the SE and QCM-D methods cannot provide independently.

The SARS-CoV-2 spike (S) protein is a large transmembrane homotrimer located on the surface of the virus. Each monomer is composed of two subunits: S1 and S2. The S1 subunit contains the receptor-binding domain (RBD), which is responsible for binding with the angiotensin-converting enzyme 2 (ACE2) receptor present on the cell surface [25]. The mutations in the RBD part are responsible for lowered antibody affinity and evasion of immune response. It is particularly important to understand the significance of S protein mutations in terms of interactions with antibodies generated by the humoral response in order to develop sensitive and selective biosensors for the detection of such antibodies. Moreover, the S protein is commonly applied in the development of various immunosensors and immunoassays for specific antibody detection, including rapid detection tests. Since the start of the COVID-19 pandemic, mutations in the S protein have been identified and compared to the reference SARS-CoV-2 genome. Currently, the five most prevalent SARS-CoV-2 variants of concern (VOCs), including Alpha (α or B.1.1.7), Beta (β or B.1.351), Gamma (P.1), Delta (B.1.617.2), and Omicron (B.1.1.529), have been identified. The Alpha variant has been found to be related to a greater rate of infection spread than other lineages, according to phylogenetic analysis [26]. Furthermore, it was linked to a greater viral load, specifically in the upper airway [27]. According to epidemiological studies, the Beta variant is approximately 50% more transmissible than previously disclosed variants [28,29]. It has been related to considerable immune evasion following vaccination and spontaneous infection, as well as lowered sensitivity to numerous monoclonal antibodies (mAbs) [30]. The SARS-CoV-2 RBD contains a core and a receptor-binding motif (RBM); the RBM mediates contacts with ACE2. The S protein RBM is a highly variable region of the RBD part that provides sentinel mutations [31,32]. Compared to wild-type (WT), SARS-CoV-2 containing RBM mutations has comparable replication effectiveness in vitro and leads to infection with similar clinical outcomes; additionally, it confers resistance to several mAbs and escapes some polyclonal antibody responses. Furthermore, a mutated S in the RBD of B.1.351 has an increased binding affinity to the ACE2 receptor [5,32]. The B.1.351 discovered in South Africa is distinguished by spike mutations, including mutations in the RBD of its S protein (N501Y, K417N, and E484K), and these mutations may lead to immune system evasion, causing widespread escape from mAbs [33]. The key to vaccine-induced protection is the ability of polyclonal antibodies to specifically interact with the SARS-CoV-2 S protein with a strong binding affinity [34].

In this study, we investigated the real-time interactions of the SARS-CoV-2 S protein of WT (SCoV2-WTS), Alpha (ScoV2-αS), and Beta (SCoV2-βS) with monoclonal antibodies against RBD (mAb-SCoV2-RBD), and convalescent human serum that contains polyclonal antibodies against the S protein (pAb-SCoV2-S) of WT. For this purpose, we merged two distinct, label-free measurement techniques: one based on changes in surface electromagnetic waves caused by the surface modification using proteins, and the other on changes in acoustic waves. This work aims to demonstrate a novel approach that shows how simultaneously combining two surface-sensitive methods for biosensing and analysis of the formed antibody layers’ mechanical and dielectric properties can provide more detailed information about the surface mass density that can be related to the ability of various antibodies to differently interact with similar structural proteins of the SARS-CoV-2 virus.

## 2. Materials and Methods

### 2.1. Materials

For this study, 11-Mercaptoundecanoic acid (11-MUA, CAS# 71310-21-9, 98%), 1-Ethyl-3-(3-dimethylaminopropyl)carbodiimide (EDC, CAS# 25952-53-8, ≥98%), N-Hydroxysuccinimide (NHS, CAS# 6066-82-6, ≥98%), sodium hydroxide (CAS# 1310-73-2, ≥97%), ethanolamine (ETA, CAS# 141-43-5, ≥99%), sodium dodecyl sulfate (SDS, CAS# 151-21-3, ≥99%), and phosphate buffered saline (PBS, pH 7.4) tablets were purchased from Sigma Aldrich. Methanol (CAS# 67-56-1, 99.9%) was purchased from Carl Roth GmbH & Co (Germany, Karlsruhe). The SARS-CoV-2 wild-type spike protein (SCoV2-WTS) and its variants (SCoV2-αS, SCoV2-βS)—expressed as secreted trimer protein in mammalian (hamster) CHO cells (>90%)—were purchased from Baltymas (Lithuania, Vilnius). QCM-D gold sensors were purchased from Biolin Scientific (Sweden, Vastra Frolundra). Human mAb-SCoV2-RBD were purchased from Abcam (UK, Cambridge).

Convalescent serum was collected from a volunteer who had been convalescent with a full dose of the Comirnaty (Pfizer, Belgium, Puurs) vaccine 3 weeks before the blood collection. Whole blood was collected in a Vacutainer tube containing a 3.5 mL CAT Serum Separator Clot Activator (Greiner Bio-One GmbH, Austria, Kremsmunster) in the laboratory of Tavo Klinika (Lithuania, Vilnius). The serum was separated after centrifugation at 5000 g for 15 min [35]. The titer of antibody against the RBD domain of the S protein in convalescent serum was determined using a chemiluminescent microparticle immunoassay and recalculated to molar concentration according to WHO recommended procedures to 137.5 nM [36]. Serum samples were stored at −20 °C until the experiment and diluted with 0.01 M of phosphate-buffered saline (PBS) solution, pH 7.4, prior to injection. Samples were collected in accordance with the Lithuanian Ethics Law. No ethics committee approval was required for this study (confirmed by the Vilnius Regional Biomedical Research Ethics Committee).

### 2.2. Measurement Setup

The experiments were performed and analyzed using a combined spectroscopic ellipsometry/quartz crystal microbalance with dissipation system (SE/QCM-D). It consists of the QCM-D QSense Explorer operating at a frequency of 5 MHz and measuring up to 7 harmonics with full viscoelastic modeling (Biolin Scientific, Sweden, Vastra Frolundra), and was connected to a rotating compensator spectroscopic ellipsometer M-2000X (J. A. Woolam, Lincoln, NE, USA). Ellipsometric measurements were performed at a fixed angle of 65° in the wavelength range of 200–1000 nm. Fluid flow was regulated with a solution injector (Cole-Parmer GmbH, Germany, Vertheim) at 1 mL/min for 60 s. The measurement chamber volume was 100 μL. After injecting all proteins into the measurement chamber, the pump was stopped, and the chamber was closed.

### 2.3. Immobilization of SCoV2-WTS, SCoV2-αS, and SCoV2-βS

The SCoV2-WTS, SCoV2-αS, and SCoV2-βS proteins were covalently immobilized on 11-MUA self-assembled monolayer (SAM) functionalized gold-coated QCM-D sensor disks. Specifically, the QCM-D gold-coated sensor disks were first rinsed with water and methanol, then immersed in methanol and placed in an ultrasonic cleaner for 3 min. After drying, the QCM-D sensor was immersed in a 1 mM solution of 11-MUA in methanol for 18 h to form a SAM. The QCM-D sensor disk functionalized with 11-MUA was inserted into a QCM-D measurement cell with optical windows for simultaneous SE analysis. Covalent immobilization of SCoV2-WTS, SCoV2-αS, or SCoV2-βS requires activation of the carboxyl groups of 11-MUA. For this purpose, a solution of 0.1 M of NHS and 0.4 M of EDC mixed in equal parts was injected into the measurement chamber for 15 min and then rinsed with PBS solution (pH 7.4). Then, 333 nM of SCoV2-WTS was injected into the measurement chamber and incubated for 60 min, followed by rinsing with PBS. A 1 M ethanolamine solution, pH 8.5, was then injected for 10 min to block all activated 11-MUA carboxyl groups.

### 2.4. Formation of Immune Complexes Using Monoclonal Antibodies and Convalescent Serum Sample

A solution of 66 nM of mAb-SCoV2-RBD in PBS solution was injected onto the SCoV2-WTS premodified sensor disk for 60 min, and the formation of the mAb-ScoV2-RBD/WTS immune complex was registered. The PBS solution was then injected for 10 min to detect any immune complex dissociation processes. A regeneration solution consisting of 50 mM of NaOH and 17.34 mM of SDS was then injected for 1 min and flushed with PBS solution for 20 min. After ensuring that the signal returned to the baseline obtained after immobilization of ScoV2-WTS and confirming the signal was stable, the convalescent serum containing pAbs-SCoV2-S was diluted with PBS solution 2.08 times (close to the concentration of mAb-SCoV2-RBD), injected into a measurement chamber for 90 min, and then washed with PBS solution. An analogous immune complex formation procedure was also used for SCoV2-αS and SCoV2-βS. After injecting all proteins for 60 s into the measurement chamber, the pump was stopped, and the chamber was closed. The interaction kinetics were established for 45–50 min in the case of injecting 66 nM of mAb-SCoV2-RBD into the PBS solution, and for 90 min in the case of diluted convalescent serum containing pAb-SCoV2-S. Because both combined measurement methods, SE and QCM-D, are only sensitive to the event that occurs at the solid–liquid interface, the diffusion process in this case does not affect the measurement of the dynamic kinetic signal.

## 3. Results and Discussion

### 3.1. Mechanical Properties of the Formed Protein Layers

**Figure 1:**
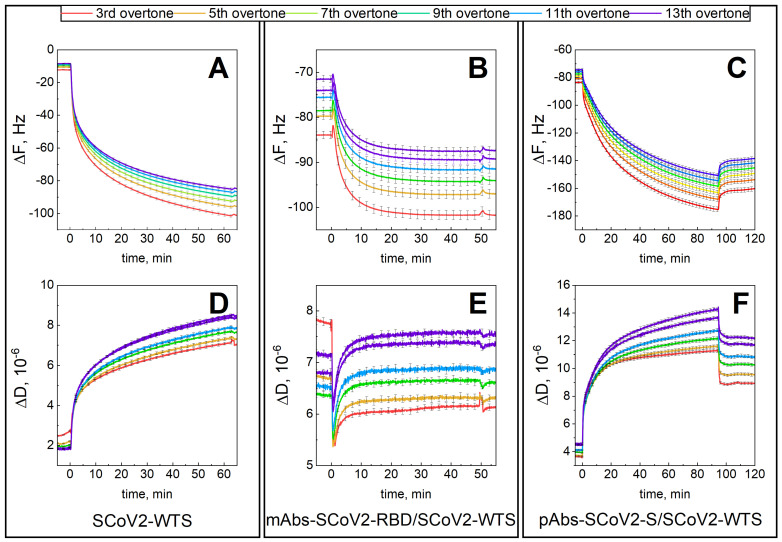
Time-resolved QCM-D kinetics of ΔF and ΔD for: SCoV2-WTS covalent immobilization on functionalized gold-coated sensor disk surface (**A**,**D**); SCoV2-WTS interaction with mAb-SCoV2-RBD antibodies (**B**,**E**), and with convalescent human serum containing pAbs-SCoV2-S antibodies (**C**,**F**).

Using a combined setup of SE and QCM-D, covalent immobilization of SCoV2-WTS on the functionalized gold-coated sensor disk surface and interaction with the mAbs-SCoV2-RBD and pAbs-SCoV2-S kinetics were registered simultaneously and are presented in Figure 1.

**Figure 2:**
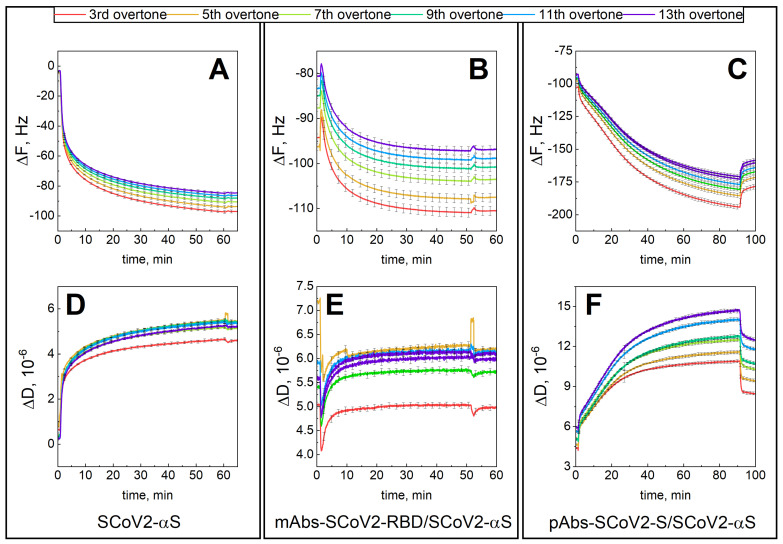
Time-resolved QCM-D kinetics of ΔF and ΔD for: SCoV2-αS covalent immobilization on functionalized gold-coated sensor disk surface (**A**,**D**); SCoV2-αS interaction with mAb-SCoV2-RBD antibodies (**B**,**E**), and with convalescent human serum containing pAbs-SCoV2-S antibodies (**C**,**F**).

The first step was covalent SCoV2-WTS immobilization of the 11-MUA functionalized surface (Figure 1A,D). This was followed by injection of a solution containing 66 nM of mAbs-SCoV2-RBD into a PBS solution. The interaction of monoclonal antibodies with SCoV2-WTS was established for 60 min and then followed by 10 min of washing with a PBS solution (Figure 1B,E). The mAbs-SCoV2-RBD monolayer was then removed by injecting regeneration solutions, and then washing it with a PBS solution. In the third step of the experiment, the convalescent human serum diluted with PBS solution 2.08 times was injected for 90 min into the measurement chamber (Figure 1C,F), and then the chamber was washed with PBS solution. The formed SCoV2-WTS layer on the functionalized gold-coated sensor disk surface expressed viscoelastic properties, as in this case, the ΔF_7_ change was 83.0 Hz (Figure 1A), and the ΔD_7_ change was 5.69 × 10^−6^ (Figure 1D), respectively. After SCoV2-WTS interaction with mAbs-SCoV2-RBD and mAbs-SCoV2-RBD/SCoV2-WTS immune complex formation, the ΔF_7_ was 15.8 Hz (Figure 1B) and ΔD_7_ was 0.9 × 10^−6^ (Figure 1E). When convalescent human serum containing pAbs-SCoV2-S was used for interaction with covalently immobilized SCoV2-WTS, the ΔF_7_ was 81.3 Hz (Figure 1C) and ΔD_7_ was 8.2 × 10^−6^ (Figure 1F), respectively. As in the case of SCoV2-WTS covalent immobilization and immune complex formation with pAbs-SCoV2-S ΔD > 1 × 10^−6^, the layers were further described as having viscoelastic properties and were characterized by applying a viscoelastic model [37]. The same experimental procedure was applied for covalent immobilization of SCoV2-αS (Figure 2A,D), which was followed by injecting a solution containing 66 nM of mAbs-SCoV2-RBD into the PBS solution and establishing interaction kinetics for 45 min (Figure 2B,E). As in the previous experiment, the final step after regeneration of the mAbs-SCoV2-RBD layer was the injection of diluted convalescent human serum for 90 min (Figure 2C,F).

Figure 2 shows the kinetics of QCM-D signals ΔF and ΔD during the formation of the SCoV2-αS layer, interaction with the mAbs-SCoV2-RBD antibodies, and formation of immune complexes with convalescent human serum containing pAbs-SCoV2-S. The change in ΔF_7_ after covalent immobilization of SCoV2-αS on the functionalized gold surface was 86.7 Hz (Figure 2A), and the formed layer also demonstrated viscoelastic properties, as ΔD_7_ was 4.7 × 10^−6^ (Figure 2D). The ΔF_7_ was 19 Hz (Figure 2B) and ΔD_7_ was 0.88 × 10^−6^ after SCoV2-αS interaction with mAbs-SCoV2-RBD, while after the immune complex formation with pAbs-SCoV2-S, the ΔF was 86 Hz (Figure 2C) and ΔD_7_ was 7.2 × 10^−6^ (Figure 2F). Thus, formed SCoV2-αS and mAbs-SCoV2-RBD layers expressed viscoelastic properties.

**Figure 3:**
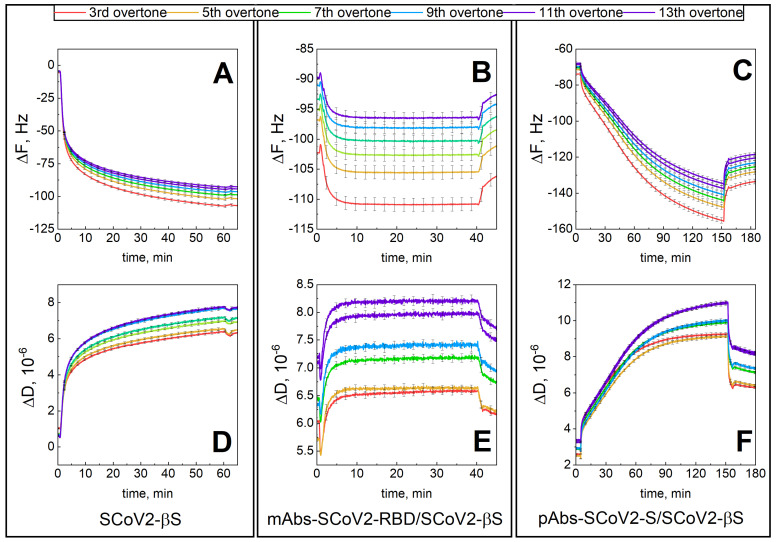
Time-resolved QCM-D kinetics of ΔF and ΔD for: SCoV2-βS covalent immobilization on functionalized gold-coated sensor disk surface (**A**,**D**); SCoV2-S interaction with monoclonal mAb-SCoV2-RBD antibodies (**B**,**E**), and with convalescent human serum containing pAbs-SCoV2-S antibodies (**C**,**F**).

The results presented in Figure 3 demonstrate the change in ΔF and ΔD during covalent immobilization of SCoV2-βS (Figure 3A,D), interaction with mAbs-SCoV2-RBD (Figure 3B,E), and with convalescent human serum containing pAb-SARS-CoV-2 (Figure 3C,F). Here, a similar experimental procedure was applied, as described in previous sections. When SCoV2-βS was successfully immobilized on the functionalized sensor disk surface, the ΔF_7_ was 76 Hz and ΔD_7_ was 6.17 × 10^−6^, respectively, demonstrating viscoelastic properties of formed layers. After the immune complex formation with mAbs-SCoV2-RBD antibodies, the ΔF_7_ was 7.7 Hz and ΔD was 1.08 × 10^−6^. In the final step, the immune complex formation was investigated between covalently immobilized SCoV2-βS, and pAbs-SCoV2-S was investigated. The results showed that ΔF_7_ was 84.4 Hz and ΔD was 6.8 × 10^−6^, expressing strong viscoelastic properties of formed layers due to the high dissipation.

**Figure 4:**
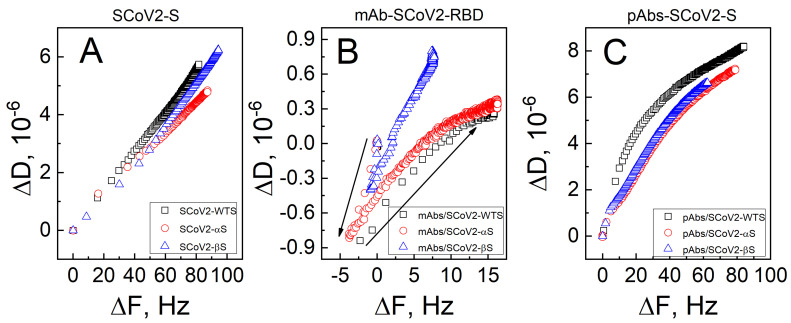
QCM-D ΔF_7_ and ΔD_7_ response to covalent SCoV-2-S proteins of different mutations immobilization on 11-MUA functionalized gold-coated sensor disk surface (**A**), followed by the immune complex formation with mAbs-SCoV2-RBD (**B**) and pAbs-SCoV2-S (**C**) antibodies.

To better evaluate viscoelastic properties of the layers caused by the conformation and structure of covalently immobilized SCoV2-WTS, SCoV2-αS, and SCoV2-βS, as well as those after immune complexes formation with mAbs-SCoV2-RBD and pAbs-SARS-CoV-2, we present ΔD/ΔF plots of the 7th harmonic in Figure 4.

As can be seen from Figure 4A, the ΔD/ΔF values for covalent immobilization of SCoV2-αS are lower than that for SCoV2-WTS and SCoV2-βS. The highest dissipation values are expressed in the case of the SCoV2-βS layer (Figure 4A, blue curve). The ΔD/ΔF ratio (induced energy dissipation per coupled mass) is commonly used in QCM-D studies to estimate the viscoelastic properties of a deposited film [38,39]. Typically, a larger ΔD/ΔF value indicates the formation of a more flexible, dissipative layer. The measured ΔD change for protein layers is mainly attributed to the orientation of asymmetric molecules during their coupling to the functionalized surface, or, in the case of antibodies, to the formation of immune complexes caused by hydrogen bonds and weak interactions between antibodies and immobilized antigens [2]. Similarly, the change in ΔD obtained analyzing the DNA layers can also be attributed to viscous drag of trapped interlayer buffer solution molecules [38]. 

To comprehend the viscoelastic properties of the formed mAbs-SCoV2-RBD layers, the ΔD/ΔF plots in Figure 4B are presented. The viscoelastic properties can be evaluated using a criterion |ΔD_n_/(ΔF_n_/n)| < 4 × 10^−7^ Hz^−1^; if this criterion is met, the layer is assumed to be rigid [40]. However, for mAbs-SCoV2-RBD interactions with SCoV2-WTS, SCoV2-αS, and SCoV2-βS, this criterion for the application of the Sauerbrey equation was not met. The mAbs-SCoV2-RBD interaction with SCoV2-WTS (Figure 4B black curve) was equal to |ΔD_7_/(ΔF_7_/7)| = |1.1 × 10^−6^/(18.1/7)| = 4.25 × 10^−7^ Hz^−1^, the mAbs-SCoV2-RBD interaction with SCoV2-αS (Figure 4B red curve)—|ΔD_7_/(ΔF_7_/7)| = |1.15 × 10^−6^/(20/7)| = 4.02 × 10^−7^ Hz^−1^, and the mAbs-SCoV2-RBD interaction with SCoV2-βS (Figure 4B blue curve)—|ΔD_7_/(ΔF_7_/7)| = |1.14 × 10^−6^/(8.62/7)| = 9.26 × 10^−7^ Hz^−1^. In the case of mAbs-SCoV2-RBD and SCoV2-βS formation of immune complexes, criterion |ΔD_n_/(ΔF_n_/n)| is about 2.3-fold higher than for immune complexes formed after the interaction of mAbs-SCoV2-RBD and SCoV2-αS. The different shape of the curves in Figure 4B reflects the change in mAb allosteric movements during the immune complex formation with mutated SCoV2-αS and SCoV2-βS.

In the case of the use of convalescent human serum containing pAbs-SCoV2-S, the ΔD/ΔF plot in Figure 4C demonstrates the highest changes after immune complex formation with covalently immobilized SCoV2-WTS (Figure 4C black curve). The pAbs-SCoV2-S antibodies’ binding-induced changes in the formed layers after interaction with SCoV2-WTS, SCoV2-αS, and SCoV2-βS is high, indicating that the pAbs-SCoV2-S antibodies had different viscoelastic properties during immune complex formation (Figure 4C). These states can be related to different classes and subclasses of pAbs-SCoV2-S antibodies and their ability to recognize different epitopes. Hence, in the case of mAb-SCoV2-RBD antibodies, more ordered layers are formed in comparison to pAbs-SCoV2-S antibodies.

### 3.2. Dielectric Properties of Formed Protein Layers

**Figure 5:**
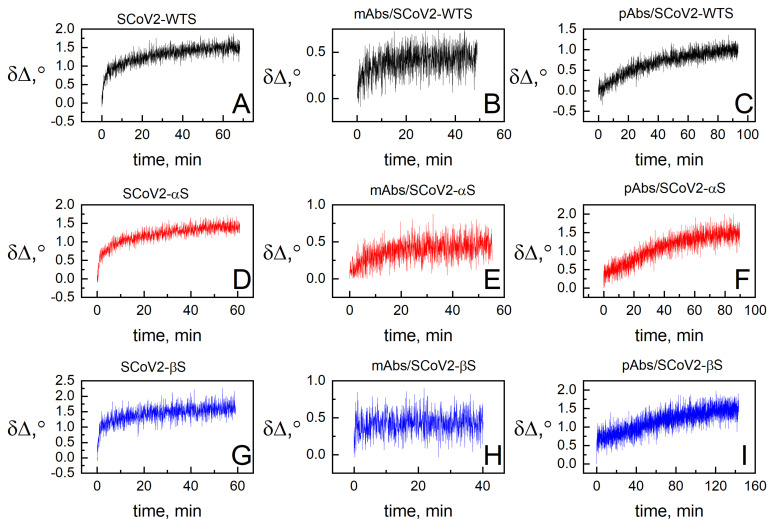
Kinetics of ellipsometric parameter δΔ: during SCoV2-WTS (**A**), SCoV2-αS (**D**), SCoV2-βS (**G**) covalent immobilization on functionalized gold-coated sensor disk surface; after immune complex formation of SCoV2-WTS (**B**), SCoV2-αS (**E**), and SCoV2-βS (**H**) with mAb-SCoV2-RBD, and pAbs-SCoV2-S antibodies (**C**,**F**,**I**), respectively.

The evolution of SE parameters in time was registered simultaneously with QCM-D measurements. In this work, we present only the ellipsometric parameter Δ changes in time due to its higher sensitivity under current experimental conditions. After covalent immobilization of SCoV2-WTS, SCoV2-αS, and SCoV2-βS (Figure 5A,D,G), the change in the ellipsometric parameter δΔ was similar and reached 1.5° after 60 min. When mAbs-SCoV2-RBD were used for interaction with SCoV2-WTS, SCoV2-αS, and SCoV2-βS, the δΔ change was 0.5° after 60 min for mAbs-SCoV2-RBD interaction with SCoV2-WTS (Figure 5B) and SCoV2-αS (Figure 5E), but in the case of SCoV2-βS (Figure 5H), the signal change was difficult to distinguish due to the low number of mAbs-SCoV2-RBD interaction with covalently immobilized SCoV2-βS. In the case of using convalescent human serum containing pAbs-SCoV2-S, the δΔ was 1.5° after pAbs-SCoV2-S interaction with immobilized SCoV2-αS (Figure 5F) and SCoV2-βS (Figure 5I), while after interaction with SCoV2-WTS (Figure 5C), it was 1.15° after 90 min, respectively.

The analysis of acoustic and electromagnetic optical signal dynamics allowed us to evaluate the surface mass density (Γ) of protein layers containing entrapped buffer solution molecules and dry protein mass that, in this case, is possible to calculate from SE data [41]. The calculation of Γ^SE^ was performed by applying the de Feijer formula [2]. The Γ^QCM-D^ were calculated using viscoelastic modeling in D-Find software [37]. The Γ^QCM-D^ were calculated using viscoelastic modeling. The wet (Γ^QCM-D^) and dry (Γ^SE^) surface mass density calculated for all stages of the protein layers’ formation are presented in Table 1.

The highest amount of dry surface mass calculated from SE measurements (Γ^SE^) equal to 382 ng/cm^2^ was obtained for SCoV2-βS covalent immobilization on the functionalized gold surface, and this value is also close to those obtained for SCoV2-WTS and SCoV2-αS. The same trend was also observed for Γ^QCM-D^: the highest value of 1739 ng/cm^2^ was obtained for covalent immobilization of SCoV2-βS. 

When immune complexes with mAbs-SCoV2-RBD were formed, the highest amount after interaction with SCoV2-WTS was calculated as Γ^SE^ = 148 ng/cm^2^. In the case of mAbs-SCoV2-RBD and SCoV2-αS interaction, Γ^SE^ = 140 ng/cm^2^ was obtained, and it was close to the results obtained using SCoV2-WTS. However, when immune complexes were formed with SCoV2-βS, the Γ^SE^ = 46 ng/cm^2^ was obtained. The same result was observed for Γ^QCM-D^ = 143 ng/cm^2^ and demonstrated lower mAbs-SCoV2-RBD ability to interact with the mutated RBD part of SCoV2-βS. The lower Γ^SE^ = 249 ng/cm^2^ and Γ^QCM-D^ = 1366 ng/cm^2^ values after pAbs-SCoV2-S immune complex formation with SCoV2-βS in comparison to SCoV2-WTS (Γ^SE^ = 297 ng/cm^2^ and Γ^QCM-D^ = 1545 ng/cm^2^) and SCoV2-αS (Γ^SE^ = 281 ng/cm^2^ and Γ^QCM-D^ = 1560 ng/cm^2^) were observed. 

### 3.3. Hydration of Formed Protein Layers

During the development of biosensors that can be applied to study real-time interaction kinetics, it is important to evaluate the composition of the formed protein layers. The hydration of the formed SCoV2-WTS, SCoV2-αS, and SCoV2-βS layers was calculated by the previously applied procedure [2,42], and the hydration values were close in range to each other. After the immune complex formation between SCoV2-βS and mAbs-SCoV2-RBD, the formed layer demonstrated the highest hydration values (67.83%) (Table 1).

The hydration and surface mass values of pAbs-SCoV2-S layers are also higher than those of mAbs-SCoV2-RBD because monoclonal antibodies interact only with the RBD part of the S protein. The remarkably lower Γ and higher hydration of formed mAbs-SCoV2-RBD and SCoV2-βS immune complexes can be related to mutations in the SCoV2-βS. The SARS-CoV-2 virus containing RBD E484K, K417N, and N501Y mutations act together and cause the escape from mAbs [33]. Due to this, the surface mass density and the hydration evaluation of the formed antibodies layer can be applied as one of the important points during the evaluation of the different classes of antibodies’ ability to neutralize the viral proteins. Other authors demonstrated that the binding affinity of SCoV2-βS RBD to the ACE2 receptor was K_D_ = 4 nM, and it is high in comparison to SCoV2-αS RBD binding to ACE2 that is 2.7-fold lower [33]. In order to successfully block the RBD part that is responsible for the ACE2 receptor attachment, the affinity of the antibody to the RBD must be higher than the RBD to the ACE2 receptor. In our previous work, we estimated the K_D_ of vaccinated human serum antibodies to SCoV2-WTS, ScoV2-αS, and ScoV2-βS, and concluded that the affinity of pAbs-SCoV2-S to all mutations was in the range lower than nanomoles [35].

## 4. Conclusions

The present study underlines how specific mechanical and optical properties of the layers formed at the solid–liquid interface can be obtained by applying two surface-sensitive, time-resolved methods. This allows quantitative evaluation of the layers’ hydration and surface mass density. The layers that consisted of the immune complex formed between SARS-CoV-2 S proteins VOCs, mAbs-SCoV2-RBD, and pAbs-SCoV2-S at the solid–liquid interface demonstrated different surface mass density and hydration values. Moreover, the calculated lower surface mass density of mAbs-SCoV2-RBD after immune complex formation with covalently immobilized SCoV2-WTS, SCoV2-αS, and SCoV2-βS in comparison to pAbs-SCoV2-S can be attributed to the fact that mAbs-SCoV2-RBD attaches only to the RBD part and has monovalent binding. On the contrary, when convalescent human serum containing pAbs-SCoV2-S is used, pAbs-SCoV2-S attaches to different parts of the S protein and recognizes different epitopes that result in higher surface mass density. The protein layers’ dielectric and viscoelastic properties play an important role in the design of label-free immunosensors. Recent findings bring insight into the variations of monoclonal and polyclonal antibody interactions with SARS-CoV-2 VOCs. Consequently, the combined optical–acoustic method can be successfully applied in the development of sensitive immunosensors and immunoanalytical systems based on monoclonal and polyclonal antibodies. 

## Figures and Tables

**Table 1:** Surface mass density (Γ) and hydration of proteins’ monolayers.

	SCoV2-S			mAb-SCoV2-RBD			pAb-SCoV2-S		
	WT	α	β	WT	α	β	WT	α	β
Γ^SE^ (ng/cm^2^)	349	358	382	148	140	46	297	281	249
Γ^QCM-D^ (ng/cm^2^)	1512	1598	1739	319	300	158	1545	1560	1366
Hydration (%)	76.92	77.59	78.03	53.60	53.33	70.88	80.78	81.99	81.77

Γ^SE^—surface mass density measured using spectroscopic ellipsometry, Γ^QCM-D^—surface mass density measured using quartz crystal microbalance with dissipation.

## Data Availability

The data presented in this study are available on request from the first author.
